# Supplementary material for: Topographic representation of current and future threats in the mouse nociceptive amygdala
Source: Nat Commun. 2023 Jan 13;14:196. doi: 10.1038/s41467-023-35826-4 (PMC9839702; doi:10.1038/s41467-023-35826-4)
Supplement: Supplementary file 1 — Supplementary Information [file 41467_2023_35826_MOESM1_ESM.pdf]

Topographic representation of current and future threats in the mouse nociceptive amygdala

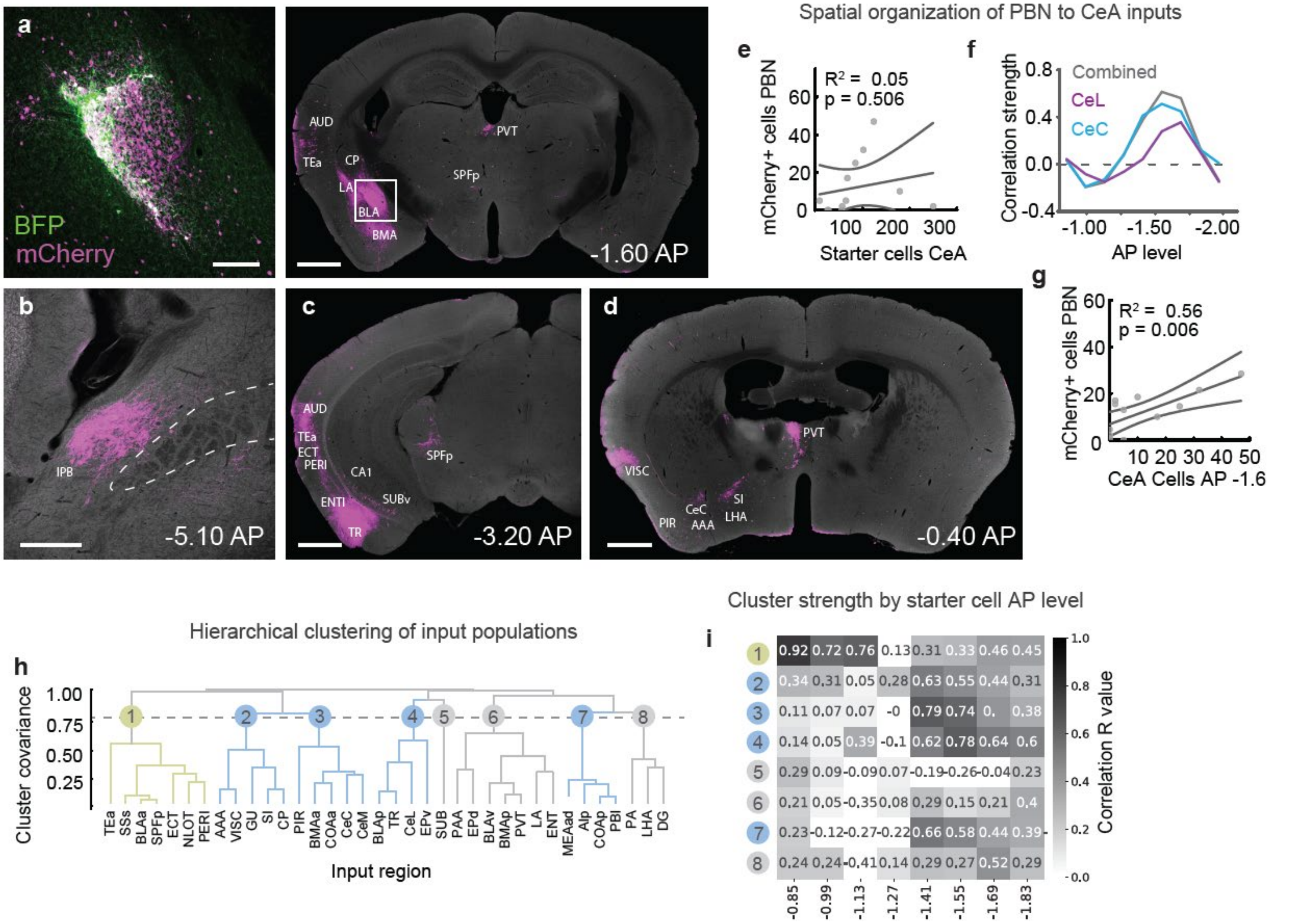

Supplementary Fig. 1 | Complementary sensory inputs to rostral and caudal CeA Calcrl+ neurons. a-d, Representative images of monosynaptically labeled retrogradely traced input neurons (mCherry) following infection of helper viruses (TVA and G-protein) in CeA Calcrl+ neurons (BFP in green). Sections were warped to common-coordinate framework space to assign segmented cells to brain regions. Injection site expression shown in **a,** (left, scale bar 100 µm). Inputs shown in **a,** (right) include CP (caudoputamen), LA (lateral amygdala), BLAa, BLAv (basolateral amygdala), BMAp (posterior basomedial amygdala), TEa (temporal association area), AUD (auditory area), PVT (periventricular thalamus), and SPFp (parvicellular subparafascicular thalamus) (scale bar 1 mm); **b**, lPB (lateral parabrachial nucleus) (scale bar 250 µm); **c**, COa (cortical amygdala), TR (postpiriform transition area), ENTl (entirhinal area), TEa, CA1, SUB (subiculum), and SPFp (scale bar 1 mm); **d**, PVT, SI (substantia innominata), LHA (lateral hypothalamic area), AAA (anterior amygdala), PIR (piriform area), and VISC (visceral area) (scale bar 1mm). **e,** Number of input neurons in PBN to CeA Calcrl+ neurons per subject not predicted well by number of overall starter cells (R^2 = 0.05, p = 0.506). **f,** Smoothened (2x neighbors) correlation between CeA Calcrl+ starter cells by AP level to total input cells captured in PBN. Input neurons in PBN best predicted by labeling starter cells located in caudal half of PBN. **g,** Number of input neurons in PBN predicted by number of Calcrl+ starter cells located at AP level -1.6 mm (slope = 0.78, R^2 = 0.56, p = 0.006). **h,** Dendrogram following hierarchical clustering based on Euclidean distance of input area cell counts cross-correlations. Resulting clusters (8 in total) based on similarity cross-correlation cutoff of 0.80. Bullet color indicates whether later correlation with starter cell location indicates relationship to rostral (green) or caudal (blue) starter cell number, or neither (grey). **i,** Results of cross-correlating cell count averages from each area in a cluster to CeA starter cells at each AP level by subject. For full statistical information see Supplementary Table 1. For brain region abbreviations see Supplementary Table 2. Related to Fig. 1.

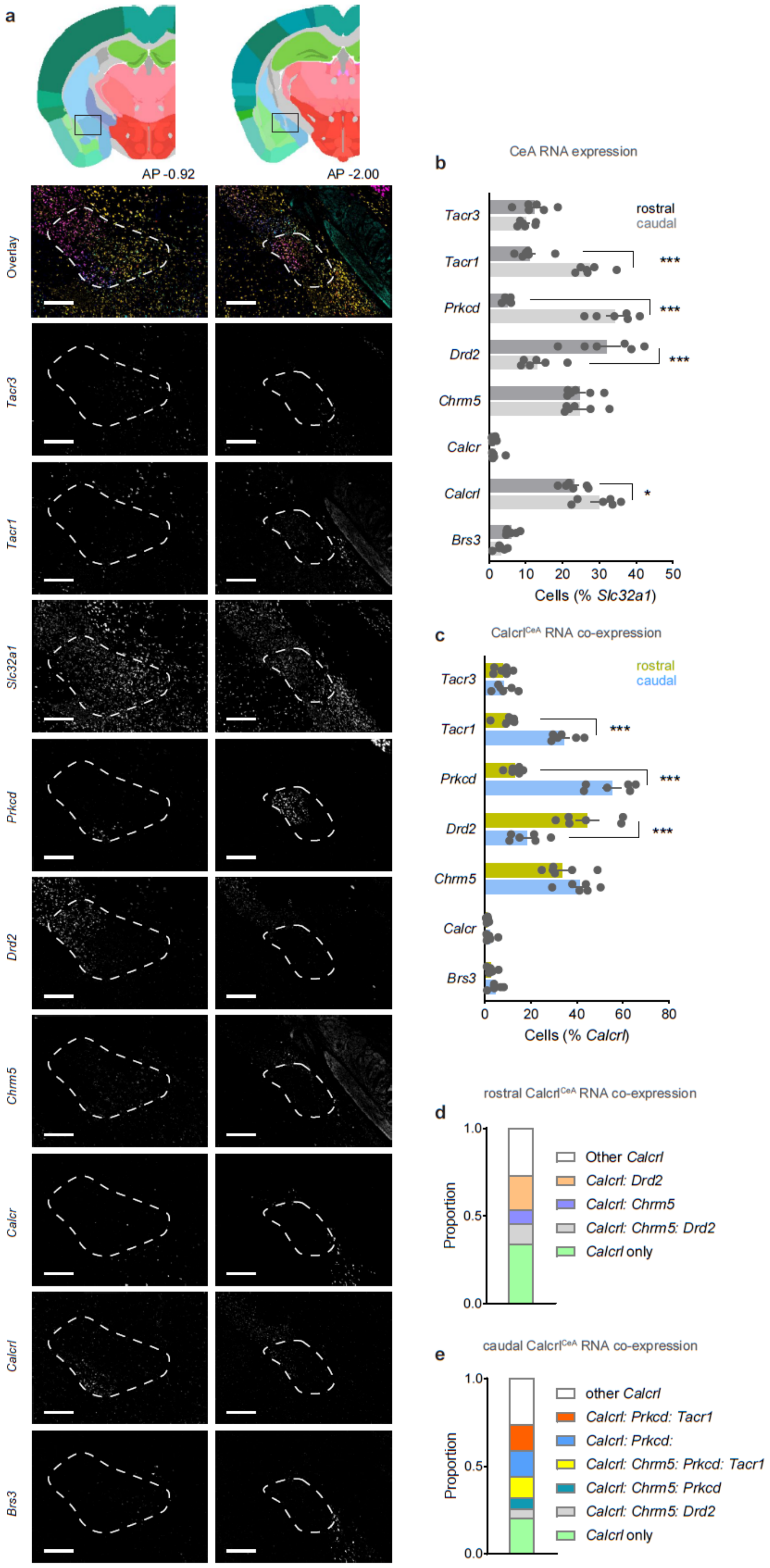

Supplementary Fig. 2 | Rostral vs caudal CeA gene expression. a, Representative images of rostral and caudal CeA coronal sections for fluorescent *in situ* hybridization. **b,** Percent of *Slc32a1* (Vgat)-expressing cells co-labeled with each probe comparing rostral and caudal sections (n=6 sections each from n=3 subjects; two-way ANOVA; Interaction: F(7, 80) = 36.26, p<0.0001; Sidak's multiple comparison.). **c,** Percent of *Calcrl*+ neurons co-labeled with each probe in rostral vs. caudal CeA (n=6 sections ea. from n=3 subjects; two-way ANOVA; Interaction: F(6, 70) = 36.22, p<0.0001; Sidak's multiple comparison.). **d,** Proportion of *Calcrl*+ neurons in the rCeA belonging to each co-expression profile. **e,** Proportion of *Calcrl*+ neurons in the cCeA belonging to each co-expression profile. *Tacr3,* tachykinin receptor 3, *Tacr1,* tachykinin receptor 1, *Slc32a1, Prkcd*, Protein kinase C-δ, *Drd2,* dopamine receptor 2, *Chrm5,* muscarinic choline receptor 5, *Calcr,* calcitonin receptor, *Brs3,* bombesin receptor subtype 3. Scale bar: 100 μm. For full statistical information see Supplementary Table 1. Related to Fig. 1.

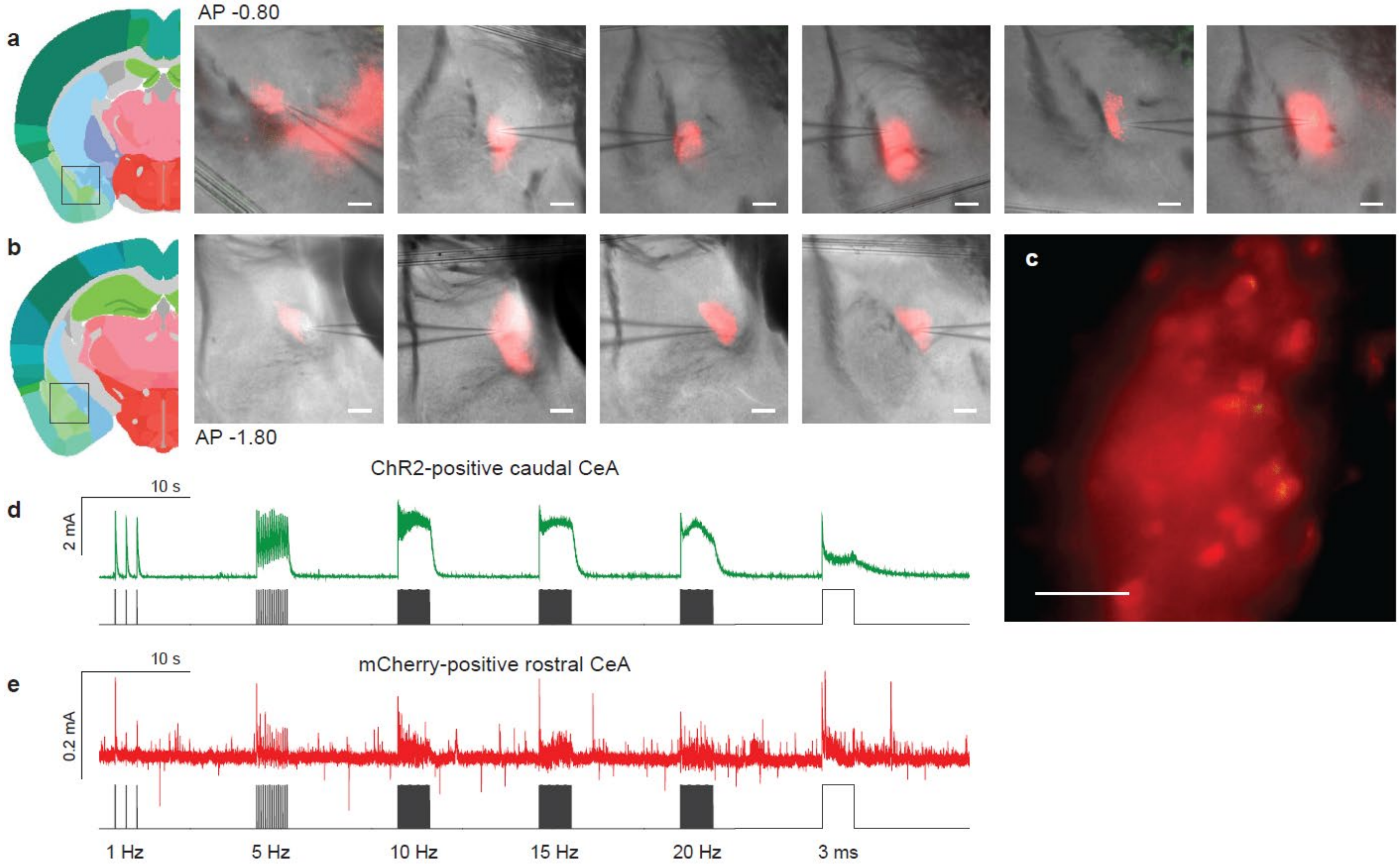

Supplementary Fig. 3 | Recording locations for slice electrophysiology. a, Representative images of coronal sections from which recordings from rostral CeA Calcrl+ neurons were obtained. For this experiment DIO-mCherry was injected at AP -0.90, DIO-ChR2-YFP was injected at AP -1.75; no ChR2-YFP spread anterior to recording site (AP -0.80). **b,** Representative images of coronal sections containing recording locations for cCeA Calcrl+ neurons. For this experiment DIO-mCherry was injected at AP -1.75, DIO-ChR2-YFP was injected at AP -0.90; no ChR2-YFP spread posterior into recording site (AP -1.80). **c,** Representative 60X image of mCherry expression absent ChR2-YFP somatic co-expression in rCeA Calcrl+ neurons (repeated in 3 animals). **d,** Example of photocurrents in ChR2-expressing cCeA Calcrl+ neuron during blue light delivery. **e,** Example of light-evoked IPSCs in an mCherry-only expressing rCeA Calcrl+ neuron. Both cells were at +10 mV holding potential. Scale bar: 100 µm.

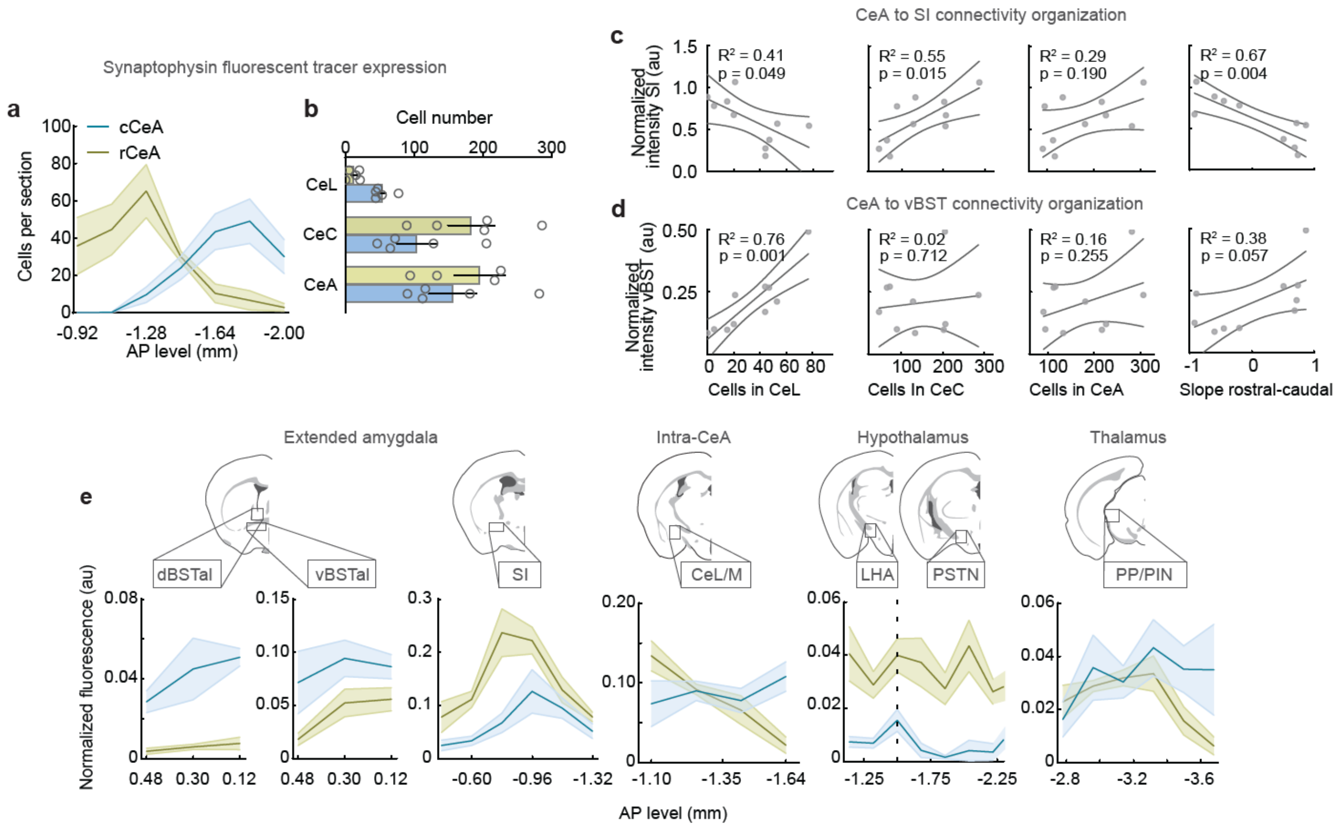

Supplementary Fig. 4 | Tracer expression patterns correlated with downstream terminal labeling.

a, Cells expressing tracer per section across the rostrocaudal axis for rostral versus caudal viral targeting (n=4 rCeA, n=5 cCeA). **b,** Summary of cell number per CeA subregion for different injection types (n=4 rCeA, n=5 cCeA). **c,** Linear regression analysis of CeA tracer-expression profiles (n=9; number of neurons in the CeL, CeC, entire CeA, or injection bias rostrocaudal for each subject) vs strength of SI synaptic labeling. Linear regression analysis of CeL to SI: R^2 = 0.41, p = 0.049; CeC to SI: R^2 = 0.55, p = 0.015; CeA to SI: R^2 = 0.29, p = 0.109; caudal bias to SI: R^2 = 0.67, p = 0.004. **d,** Linear regression analysis of CeA tracer expression profiles vs strength of ventral alBST synaptic labeling. Linear regression of CeL to vBSTal: R^2 = 0.76, p = 0.001; CeC to vBSTal: R^2 = 0.02, p = 0.712; CeA to vBSTal: R^2 = 0.16, p = 0.255; caudal bias to vBSTal: R^2 = 0.38, p = 0.057. Bands around regression line: 95% confidence interval for regression slope. **e,** Fluorescence in downstream targets relative to total projection intensity. Data represented as mean±SEM. For full statistical information see Supplementary Table 1. Related to Fig. 2.

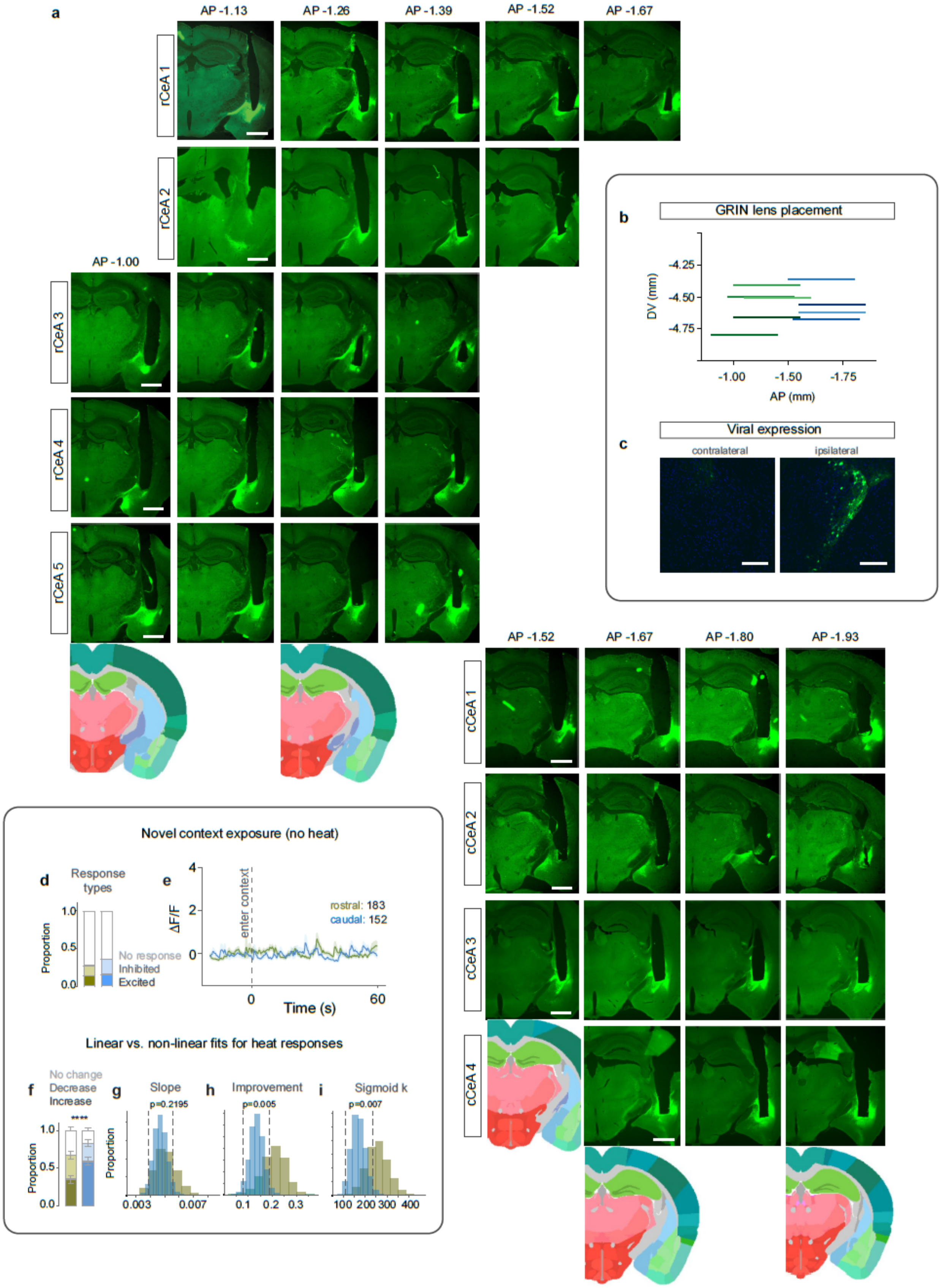

Supplementary Fig. 5 | GRIN lens placement for 1-photon Ca imaging experiments and hotplate experiment controls. a, Images of 600 µm diameter GRIN lens placement for rostral (top 5) and caudal (bottom 4) targeting cohorts. Coronal images of lens tip are arranged anterior (left) to posterior (right), sections separated by 130 µm (scale bar: 1mm). **b,** Summary of full lens tip footprint (AP/DV) for each subject in rostral vs caudal insertion groups. **c,** GFP expression in DIO-GCaMP6m-expressing vs. contralateral CeA in Calcrl-Cre:GFP mouse (scale bar: 100 µm). **d,** Proportion of CeA Calcrl+ neurons responding to exposure to a novel context without noxious heat. **e,** Population-averaged calcium activity during home cage vs. first 60-s in a novel context. **f,** Proportion of heat-excited

CeA Calcrl+ neurons with significant positive or negative slope during first 60-s of hotplate exposure (corresponding to a linear change in activity with length of heat exposure). Bootstrapped means and standard deviations for bars and error; two-way ANOVA; Interaction: $F(2, 21) = 38.13$, $p<0.0001$; Sidak's multiple comparison. **g,** Bootstrapped distributions of population averages for slope from Calcrl+ neurons with linearly increasing heat encoding (left), corresponding to dark bars from **(f)**. **h,** Reduction in standard error when switching from linear to sigmoid function to fit heat-evoked activity for rCeA vs cCeA Calcrl+ neurons. Bootstrapped distributions of population averages; Student's two-sided t-test $p=0.0047$. **i,** Maximum slope of sigmoid function (*k*) generated from least-squares optimized fit of heat-evoked activity in rCeA vs cCeA Calcrl+ neurons. Bootstrapped distributions of population averages; Student's two-sided t-test $p=0.007$. Related to Figs. 3, 5 and 6.

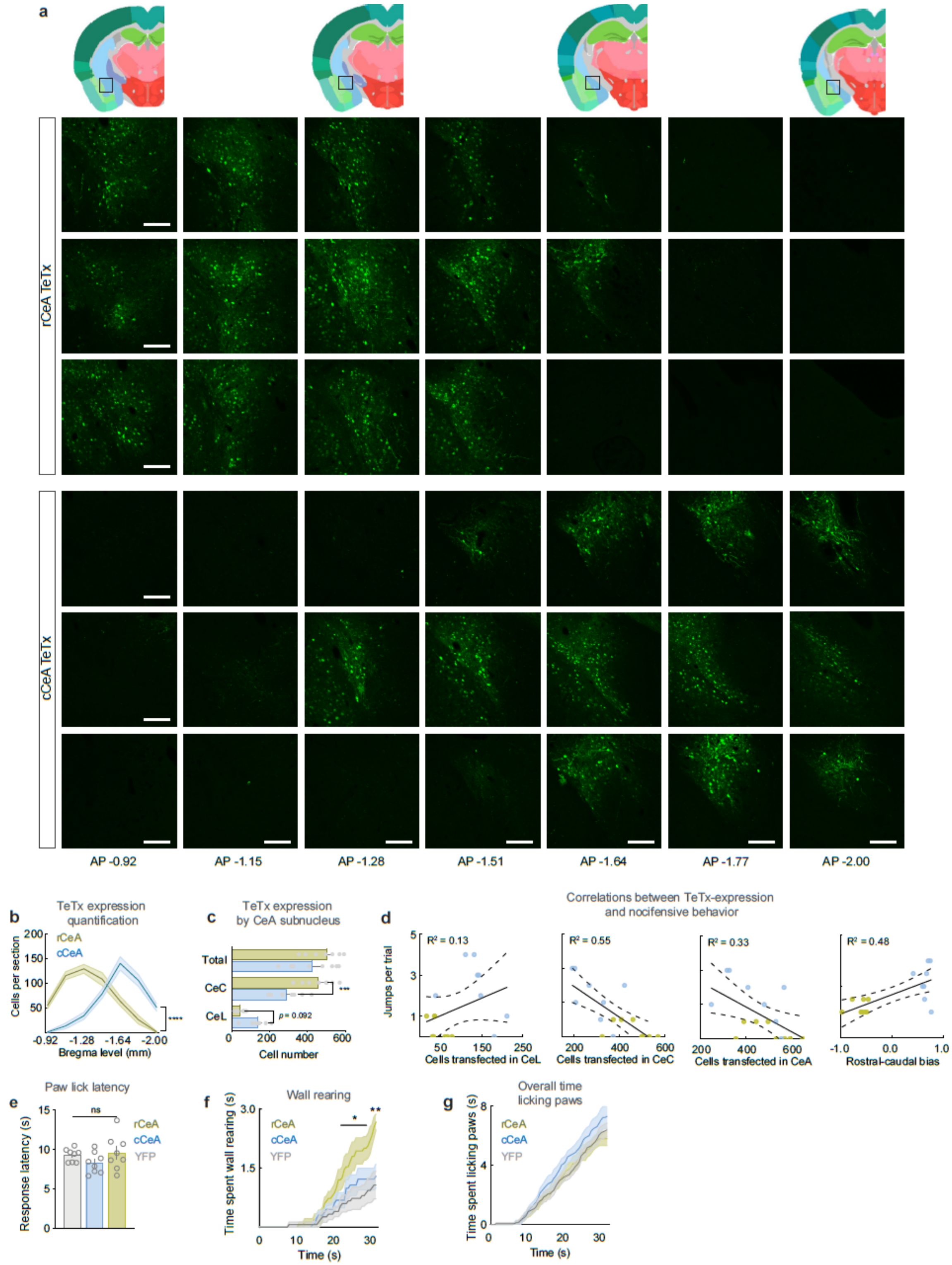

Supplementary Fig. 6 | Involvement of CeA Calcrl+ neurons in nocifensive behaviors. **a,** Example images of TeTx expression following selective injections into the rCeA (top 3 rows) or cCeA (bottom 3 rows). For this experiment, 140 nl of DIO-TeTX:GFP was injected at AP -0.90 for rCeA-expression group or AP -1.75 for cCeA expression group (scale bar: 100 µm). **b,** Summary of expression across CeA following rostral or caudal targeted injections (two-way ANOVA, Interaction: $F(6, 98) = 40.53$, $p<0.0001$). **c,** Quantification of cells transduced in CeA subnuclei from each injection type (two-way ANOVA, Interaction: $F(2, 42) = 9.99$, $p = 0.0003$; Sidak's multiple comparison). **d,** Correlations between CeA TeTx-expression profiles (number of cells per subregion or rostral vs caudal expression bias) and jumping behavior on 57°C hot plate. R-squared for each linear relationship is listed in upper left corner of each graph. Jumping behavior for each subject best predicted by number of cells transduced with TeTx in rostral CeA or CeC. e, Paw-lick latency was unaffected in mice with silenced CeA Calcrl+ neurons tested on 57°C hot plate (n = 8 per group, one-way ANOVA, $F(2, 21) = 1.50$, $p = 0.246$). **f,** Silencing rCeA Calcrl+ neurons enhanced time spent wall rearing (two-way RM-ANOVA, Interaction: $F(118, 1180) = 4.05$, $p<0.0001$; Tukey's multiple comparison). **g,** Silencing CeA Calcrl$^+$ neurons did not affect cumulative time spent paw licking during 57°C hot-plate test (n = 8 each group; two-way RM ANOVA, Interaction: $F(118, 1180) = 1.10$, $p = 0.234$). Data represented as mean±SEM. *$p < 0.05$; **$p < 0.01$; ***$p < 0.001$; ****$p < 0.0001$. For full statistical information see Supplementary Table 1. Related to Fig. 3 and 5.

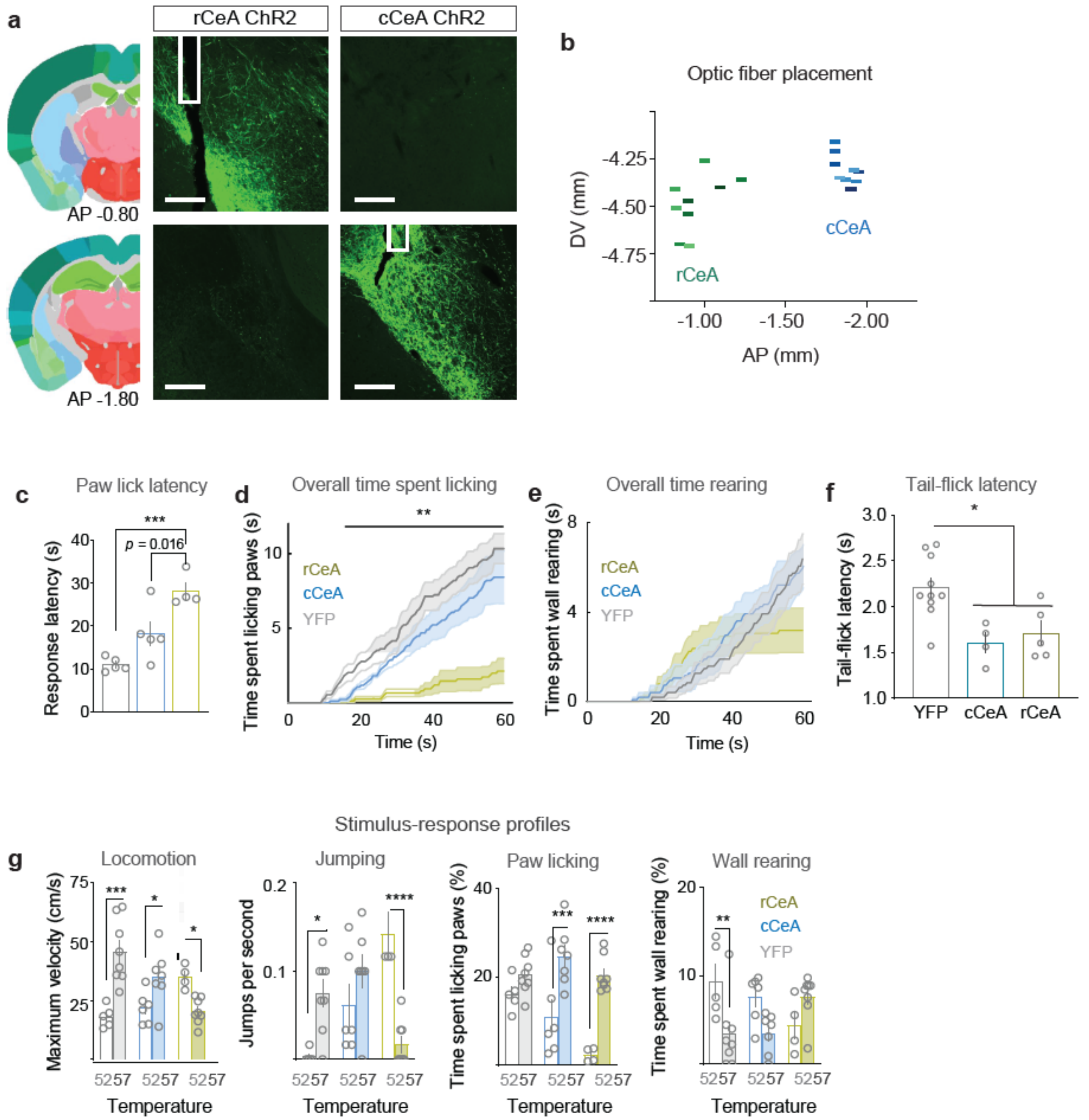

Supplementary Fig. 7 | Effect of photostimulation of CeA Calcrl+ neurons on nocifensive behavior. a, Images of optic fiber placement and expression of DIO-ChR2-YFP following injection/fiber insertion at AP -0.90 for rostral group or AP -1.75 for caudal group (repeated 9 times for each group, scale bar: 100 µm). **b,** Summary of optic fiber tip locations (AP/DV) in rostral (n=9) or caudal (n=9) targeted groups. **c,** Photostimulation of rCeA Calcrl+ neurons delayed initial response to 52°C hot plate (n = 5 control, 4 rostral, 5 caudal; one-way ANOVA, $F(2, 11) = 17.08$, $p = 0.0004$; Tukey's multiple comparison) and **d,** reduced cumulative time spent licking paws (right) (two-way RM-ANOVA, Interaction: $F(226, 1243) = 8.283$, $p<0.0001$; Tukey's multiple comparison). **e,** CeA Calcrl$^+$ neuron photostimulation had no effect on wall rearing during the hot-plate test. **f,** Effect of CeA Calcrl$^+$ neuron photostimulation on tail-flick latency (measure of spinal algesia, 52 °C water bath) (n = 10 control, 5 rostral, 4 caudal; one-way ANOVA $F(2, 16) = 7.48$, $p = 0.0051$; Tukey's multiple comparison). **g,** Stimulus-response profiles in experimental groups at 52°C (open bars) or 57°C (closed bars) (two-way ANOVA with Sidak's multiple comparison; Locomotion – Interaction: $F(2, 32) = 15.33$, $p<0.0001$; Jumping – Interaction: $F(2, 32) = 17.50$, $p<0.0001$; Paw licking – Interaction: $F(2, 32) = 4.44$, $p = 0.020$; Wall rearing – Interaction: $F(2, 32) = 6.49$, $p = 0.004$; Tukey's multiple comparison). Data represented as mean±SEM. *$p < 0.05$; **$p < 0.01$; ***$p < 0.001$; ****$p < 0.0001$. For full statistical information see Supplementary Table 1. Related to Fig. 3-6.

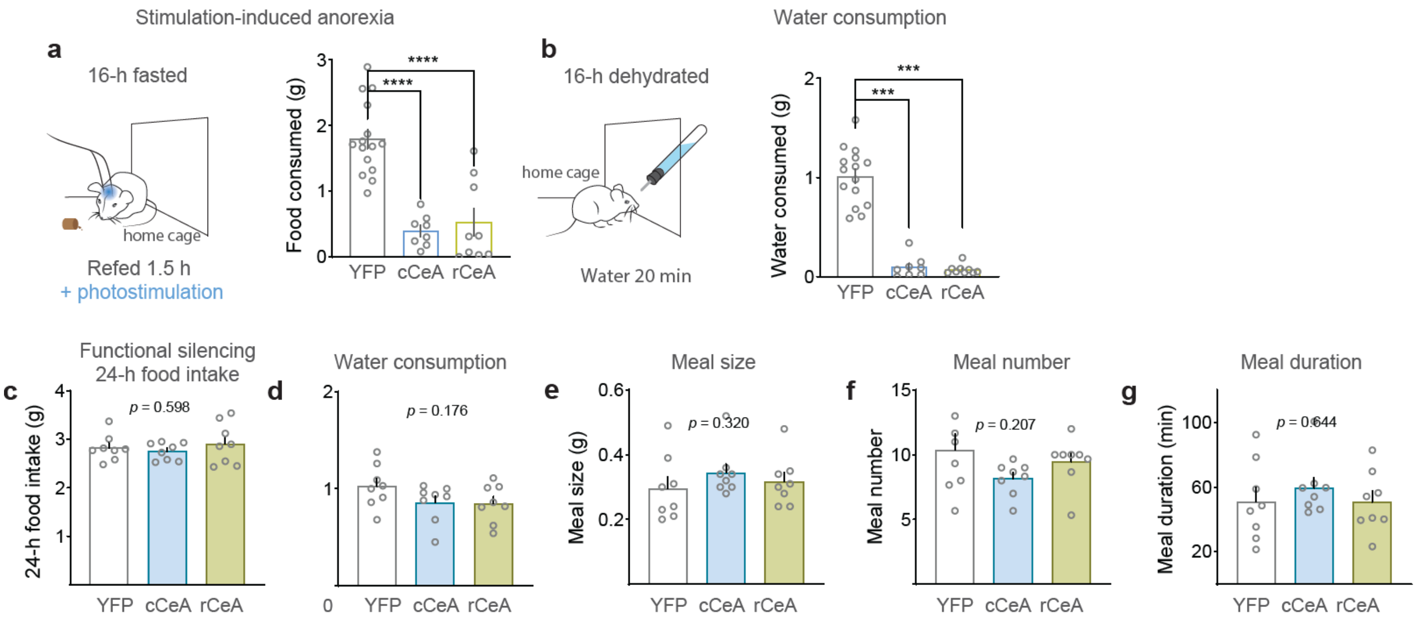

Supplementary Fig. 8 | Rostral and caudal CeA Calcrl+ neurons both potently suppress ingestion, but neither are required for normal satiety. a, 1.5-h food intake in fasted mice with photostimulation of either rCeA or cCeA Calcrl$^+$ neurons; photostimulation potently suppresses appetite (n=15 control, 9 caudal, 9 rostral; one-way ANOVA $F(2, 29) = 25.97$, $p<0.0001$; Tukey's multiple comparison). **b,** Photostimulation of either rostral or caudal CeA Calcrl$^+$ neurons potently suppressed water intake in thirsty mice (n=15 control, 9 caudal, 9 rostral; Kruskal-Wallis test, $p<0.0001$; Dunn's multiple comparison). **c,** 24-h food intake is unaltered in mice with silenced CeA Calcrl$^+$ neurons (n=8 each group; one-way ANOVA $F(2, 21) = 0.528$, $p=0.598$). **d,** 20-min water consumption in 16-h dehydrated mice with silenced CeA Calcrl$^+$ neurons (n=8 each group; one-way ANOVA, $F(2, 21) = 1.89$, $p = 0.176$). **e,** Average meal size over 24 h in mice with silenced CeA Calcrl+ neurons is unaffected (n=8 each group; Kruskal-Wallis test, $p = 0.320$). **f,** Cumulative meal number (over 24-h) in mice with silenced CeA Calcrl+ neurons is unaffected (n=8 each group; one-way ANOVA, $F(2, 21) = 1.70$, $p = 0.207$). **g,** Average meal duration over 24 h in mice with silenced CeA Calcrl+ neurons is unaffected (n=8 each group; one-way ANOVA, $F(2, 21) = 0.449$, $p = 0.644$). Data represented as mean±SEM. ***$p < 0.001$; ****$p < 0.0001$. For statistical information see Supplementary Table 1. Related to Fig. 3.

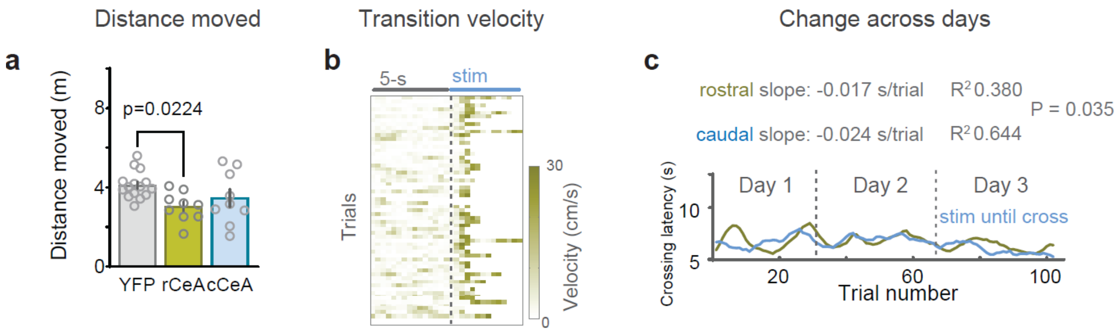

Supplementary Fig. 9 | CeA Calcrl+ neurons promote operant avoidance behavior. a, Stimulation of rCeA Calcrl+ neurons in the RTPA test reduces locomotor behavior (n = 15 control, 9 rostral, 9 caudal; one-way ANOVA, F(2, 30) = 4.19, p = 0.0248; Tukey's multiple comparison). **b,** Heatmap of an example mouse's velocity when crossing sides to avoid rCeA Calcrl+ neuron stimulation. **c,** Crossing latency across training days and trials for mice trained to avoid either rCeA or cCeA photostimulation (linear regression analysis; slope of group-average, smoothed performance on trials across days 2 and 3; rCeA slope = -0.017, R^2 = 0.38, p < 0.0001; cCeA slope = -0.24, R^2 = 0.64, p < 0.0001 that slopes=0; slope comparison: one-way ANOVA F(2, 158) = 4.509, p = 0.035). Data represented as mean±SEM. For full statistical information see Supplementary Table 1. Related to Fig. 4.

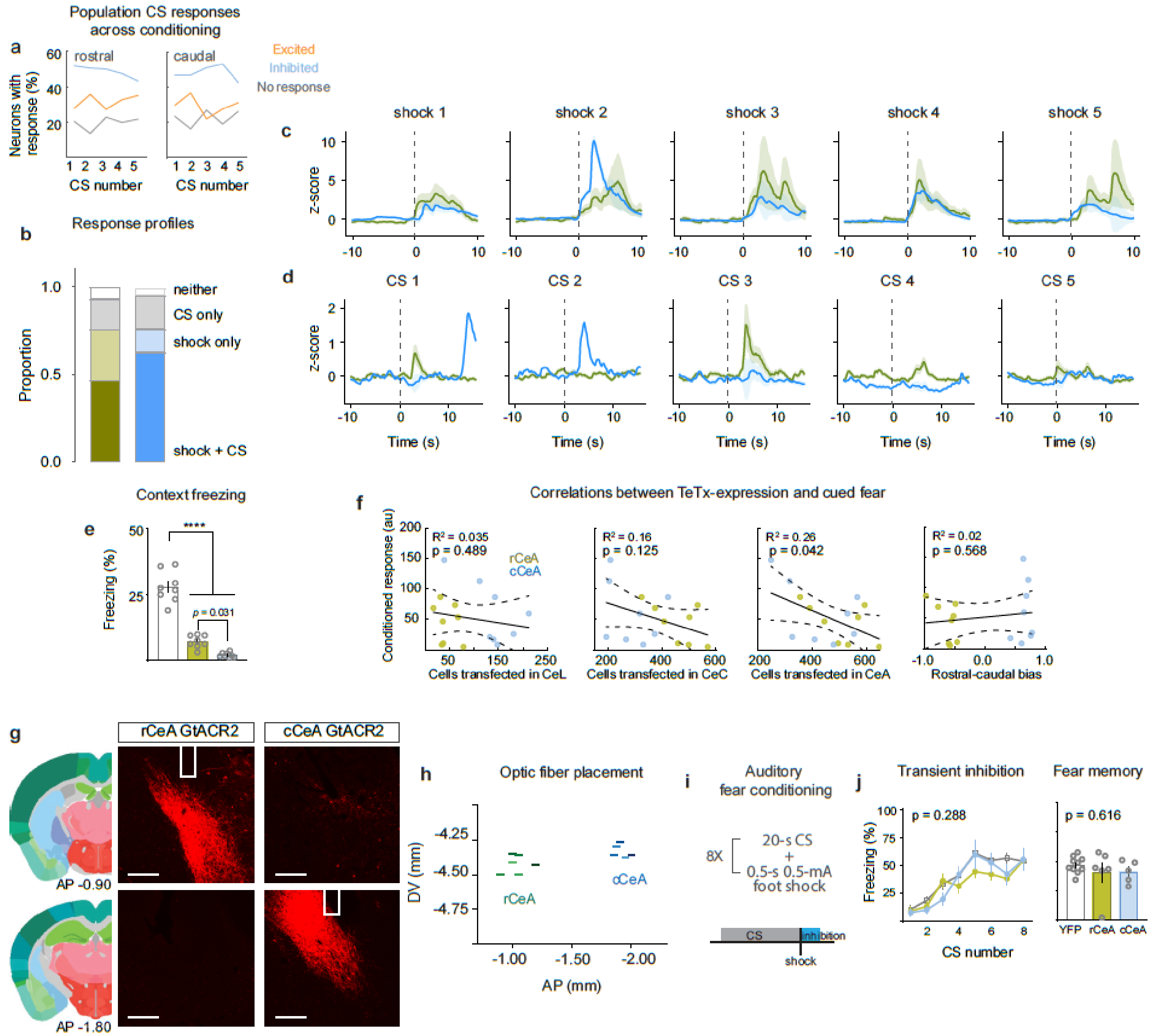

Supplementary Fig. 10 | CeA Calcrl+ neuron activity during fear learning. a, Proportion of neurons in rCeA and cCeA with significant changes in activity during each CS across auditory fear conditioning. **b,** Proportion of CeA Calcrl+ neurons with responses to auditory (CS) or somatic (shock) stimuli during fear conditioning paradigm. **c,** Population average activity during foot shock across the 5 conditioning trials (from n=183 rCeA, n=128 cCeA). **d,** Population average activity during first 15-s of CS across the 5 conditioning trials (from n=183 rCeA, n=128 cCeA). **e,** Functional silencing of CeA Calcrl+ neurons attenuates contextual fear memory (tested in conditioning context 24-h after shock exposure) (one-way ANOVA with Sidak's multiple comparison, $p<0.0001$). **f,** Linear regression between CeA TeTx-expression profiles (number of cells per subregion or rostral vs caudal expression bias) and strength of conditioned-fear responses (area under the curve for cue-evoked freezing during conditioning). CeL TeTx and fear memory: R^2 = 0.04, p = 0.489; CeC TeTx and fear memory: R^2 = 0.16, p = 0.125; CeA TeTx and fear memory: R^2 = 0.26, p = 0.042; TeTx spatial bias and fear memory: R^2 = 0.02, p = 0.568. Bands around regression lines indicate limits of 95% confidence interval for regression slope. **g,** Images of optic fiber placement and expression of DIO-GtACR2-mCherry following injection/fiber insertion at AP -0.90 for rostral group or AP -1.75 for caudal group (repeated 6 times for rostral group and 5 times for caudal group, scale bar: 100 µm). **h,** Summary of optic fiber tip locations (AP/DV) in rostral (n=6) or caudal (n=5) targeted groups. **i,** Schematic of fear conditioning paradigm to test effect of transient inhibition of CeA Calcrl+ neurons on fear acquisition. **j,** Transient inhibition of CeA Calcrl+ neurons during foot shock delivery has no effect on fear acquisition (n=10 control, 6 rostral, 5 caudal; 2-way RM ANOVA; Interaction: $F(14, 126)$ = 1.193, p = 0.2888) or on conditioned freezing behavior tested 24-h later (Browne-Forsythe ANOVA, $F(2, 8.21)=0.512$, $p=0.616$). Data represented as mean±SEM. *p < 0.05; **p < 0.01; ***p < 0.001; ****p < 0.0001. Across group comparisons ##p < 0.01; ####p < 0.0001. For full statistical information see Supplementary Table 1. Related to Fig. 5.

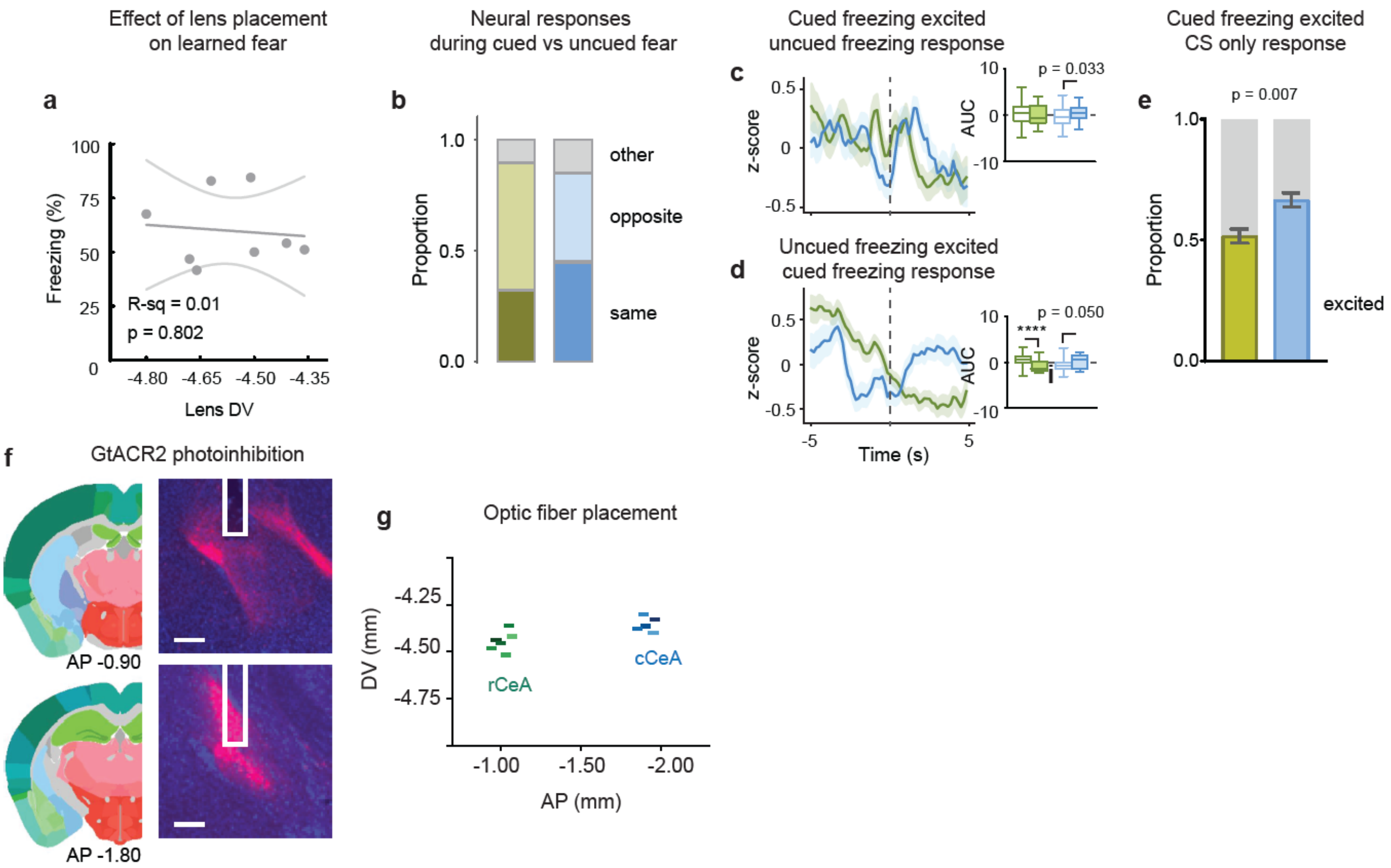

Supplementary Fig. 11 | CeA Calcrl+ neuron activity during fear memory recall. a, CeA damage caused by GRIN lens placement depth had no effect on fear acquisition in imaging subjects (bands around line indicate limits of 95% confidence interval for regression slope). **b,** Proportion of CeA Calcrl+ neurons with same or opposite responses during CS or spontaneous freezing behavior (e.g., both excited vs excited during cued freezing but inhibited during uncued freezing). **c,** Averaged activity of CeA Calcrl+ neurons that are excited during cued freezing behavior, during uncued freezing (two-way RM ANOVA; Interaction: $F(1, 130) = 5.326$, $p = 0.0226$; Sidak's multiple comparison; centre at median, box bounds 25th and 75th percentiles, whiskers minima and maxima). **d,** Averaged activity of CeA Calcrl+ neurons that are excited during uncued freezing behavior, during cued freezing (two-way RM ANOVA; Interaction: $F(1, 151) = 24.65$, $p < 0.0001$; Sidak's multiple comparison; centre at median, box bounds 25th and 75th percentiles, whiskers minima and maxima). **e,** Proportion of cued-freezing excited CeA Calcrl+ neurons that maintain their activity during the CS in the absence of freezing behavior (bootstrapped means and confidence interval, Student's two-sided unpaired t-test, p = 0.007). **f,** Images of optic fiber placement and expression of DIO-GtACR2-mCherry following injection/fiber insertion at AP -0.90 for rostral group or AP -1.75 for caudal group (scale bar: 100 µm). **g,** Summary of optic-fiber tip locations (AP/DV) in rostral (n=6) or caudal (n=5) targeted groups. Data represented as mean±SEM. ****p < 0.0001. For full statistical information see Supplementary Table 1. Related to Fig. 6.

Supplementary Table 1

Figure 1								
Panel	Experiment	N (ct/exp)	Test	Test statistics	P-values	Post-test	95% CIs	P-values
h	BLA-CeA retrograde tracing	12	Wilcoxan signed rank test	Sum of signed ranks: 78.00	P=0.0005			
j (top left)	CeC-CeC Calcrl conn	12	linear regression	Slope: 1.135; Int: 118; R-sq: 0.132	P=0.2457		-0.9157 to 3.186	
j (mid left)	CeL-CeC Calcrl conn	12	linear regression	Slope: 1.624; Int: 121.1; R-sq: 0.363	P=0.0372		0.1177 to 3.130	
j (bot left)	SI-CeC Calcrl conn	12	linear regression	Slope: 0.838; Int: 15.03; R-sq: 0.523	P=0.0078		0.2746 to 1.402	
j (top right)	CeC-CeL Calrl conn	12	linear regression	Slope: 1.888; Int: 91.27; R-sq: 0.341	P=0.0460		0.04038 to 3.735	
j (mid right)	CeL-CeL Calcrl conn	12	linear regression	Slope: 2.445; Int: 92.63; R-sq: 0.776	P=0.0002		1.519 to 3.372	
j (bot right)	SI-CeL Calcrl conn	12	linear regression	Slope: 0.674; Int: 22.75; R-sq: 0.316	P=0.0569		-0.02400 to 1.372	
l	Calcrl interconnectivity	41 neurons, 5 mice	Mann Whitney test	Mann-Whitney U: 80	P=0.0001			
m	cCeA->rCeA IPSC amplitude	20 neurons, 3 mice	One sample Wilcoxan	Sum of pos|neg ranks: 91.00|0.00	P=0.0002			

Figure 2								
Panel	Experiment	N (ct/exp)	Test	t/F (DFn, DFd)	P-values	Post-test	95% CIs	P-values
d	Anterograde tracing	5 ea	2way ANOVA, Sidak's MC	Interaction: F (7, 63) = 20.52	P<0.0001	PP/SPFp (caudal v rostral injection)	-0.2666 to 12.49	0.0684
				Injection: F (1, 63) = 0.05974	P=0.8077	PSTN	-13.77 to -1.006	0.0140
				Area: F (7, 63) = 94.62	P<0.0001	LHA	-10.35 to 2.414	0.5058
						CeAlm	-1.668 to 11.09	0.2860
						SI	-26.63 to -13.87	<0.0001
						cBST	-3.294 to 10.24	0.7340
						dBSTal	1.336 to 14.10	0.0090
						vBSTal	4.785 to 17.55	<0.0001

Figure 3								
Panel	Experiment	N (ct/exp)	Test	t/F (DFn, DFd)	P-values	Post-test	95% CIs	P-values
d	Response area under curve rostral	82 neurons	One sample Wilcoxan (0 median)	Discrepancy: 262.6	P<0.0001			
	Response auc caudal	83 neurons	One sample Wilcoxan (0 median)	Discrepancy: 284.1	P<0.0001			
g (left)	R-squared for time-increasing	10000 ea. (bootstrap)	Unpaired ttest		P = 0.0010			
j	Jumps TeTx	8 ea.	one-way ANOVA, Tukey's MC	F (2, 21) = 7.374	P=0.0038	YFP vs. cCeA	-1.771 to 1.021	0.7791
						YFP vs. rCeA	0.2294 to 3.021	0.0207
						cCeA vs. rCeA	0.6044 to 3.396	0.0045
m	Jumps ChR2	5 ct, 5 caudal 4 rostral	one-way ANOVA, Dunnett's MC	F (2, 11) = 14.07	P=0.0009	YFP vs. rCeA	-13.06 to -4.544	0.0005
						YFP vs. cCeA	-6.813 to 1.213	0.18
n	Jumps during stim rostral	4	Paired t-test	t=3.894, df=3	P=0.03			
q (left)	LPS excitation caudal	64	Wilcoxan Signed Rank Test	0 min	0.0986			
				30 min	<0.0001			
				60 min	<0.0001			
				90 min	0.0002			
				120 min	0.16			
				150 min	0.2613			
q (right)	LPS excitation rostral	57	Wilcoxan Signed Rank Test	0 min	0.1016			
				30 min	0.0274			
				60 min	0.0002			
				90 min	<0.0001			
				120 min	<0.0001			
				150 min	<0.0001			
s	LPS anorexia TeTx	8 ea.	one-way ANOVA, Tukey's MC	F (2, 21) = 6.679	P=0.0057	YFP vs. cCeA	-43.25 to -6.688	0.0066
						YFP vs. rCeA	-23.05 to 13.51	0.7898
						cCeA vs. rCeA	1.914 to 38.48	0.0287

Figure 4								
Panel	Experiment	N (ct/exp)	Test	t/F (DFn, DFd)	P-values	Post-test	95% CIs	P-values
b	RTPA	15 ct, 9 r, 9 c	one-way ANOVA, Tukey's MC	F (2, 30) = 59.64	P<0.0001	YFP vs. cCeA	26.01 to 47.44	<0.0001
						YFP vs. rCeA	30.93 to 52.36	<0.0001
						cCeA vs. rCeA	-7.062 to 16.90	0.5752
c	Memory (24-h post)	5, 5 caudal, 4 rostral	one-way ANOVA, Tukey's MC	F (2, 11) = 16.99	P=0.0004	YFP vs. cCeA	27.48 to 74.95	0.0003
						YFP vs. rCeA	1.043 to 51.39	0.0413
						cCeA vs. rCeA	-50.17 to 0.1771	0.0516
d (right)	Difference RTPP:Memory	5, 5 caudal, 4 rostral	one-way ANOVA, Tukey's MC	F (2, 11) = 5.376	P=0.0235	YFP vs. cCeA	-13.19 to 29.30	0.5780
						YFP vs. rCeA	-41.43 to 3.638	0.1036
						cCeA vs. rCeA	-49.48 to -4.414	0.0202
g	Active avoidance crossing latency	5, 5 caudal, 4 rostral	Kruskal-Wallis, Dunn's MC	Kruskal-Wallis statistic: 25.97	<0.0001	YFP vs. cCeA	23.58	<0.0001
						YFP vs. rCeA	24.02	<0.0001
						cCeA vs. rCeA	0.4375	>0.9999
h (left)	Velocity first 25 trials	5, 5 caudal, 4 rostral	Mixed-effects model, Tukey's MC	Interaction: F (36, 2554) = 4.459	<0.0001			
				Time: F (6.896, 978.5) = 5.923	<0.0001			
				Group: F (2, 142) = 1.072	0.345			
h (right)	Velocity last 25 trials	5, 5 caudal, 4 rostral	2way RM ANOVA, Tukey's MC	Interaction: F (38, 3002) = 37.93	P<0.0001			
				Time: F (4.581, 723.7) = 85.93	P<0.0001			
				Group: F (2, 158) = 45.72	P<0.0001			
i (right)	Early vs. late Velocity stim	5, 5 caudal, 4 rostral	2way RM ANOVA, Sidak's MC	Interaction: F (1, 7) = 0.7480	P=0.4158	rCeA: Early vs. Late	-7.678 to -1.447	0.0086
				Time: F (1, 7) = 4.841	P=0.0637	cCeA: Early vs. Late	-6.073 to -0.4991	0.0247
				Group: F (1, 7) = 28.28	P=0.0011			

Figure 5								
Panel	Experiment	N (ct/exp)	Test	t/F (DFn, DFd)	P-values	Post-test	95% CIs	P-values
e	Footshock excited AUC	42 caudal, 62 rostral	2way RM ANOVA, Tukey's MC	Interaction: F (2, 204) = 3.527	P=0.0312	Caudal Pre vs. shock 2	-3.356 to 1.399	0.5805
				Time: F (1.486, 151.6) = 127.3	P<0.0001	Caudal Pre vs. shock 8	-11.66 to -8.306	<0.0001
				Group: F (1, 102) = 4.291	P=0.0408	Caudal shock 2 vs. shock 8	-11.69 to -6.315	<0.0001
						Rostral Pre vs. shock 2	-7.195 to -2.408	<0.0001
						Rostral Pre vs. shock 8	-14.11 to -10.74	<0.0001
						Rostral shock 2 vs. shock 8	-10.67 to -4.572	<0.0001
f	Auto-correlations footshock 1 responses	42 caudal, 62 rostral	2way RM, Sidak's MC	Interaction: F (4, 555) = 3.965	P=0.0035	Shock 2: cCeA vs rCeA	-0.5840 to -0.1754	<0.0001
				Time: F (4, 555) = 81.49	P<0.0001	Shock 3: cCeA vs rCeA	-0.5151 to -0.1066	0.0005
				Area: F (1, 555) = 28.03	P<0.0001			
h	TeTx shock reactivity	8 ea.	one-way ANOVA, Dunnett's MC	F (2, 21) = 3.909	P=0.0360	YFP vs. cCeA	-3.981 to 21.37	0.2040
						YFP vs. rCeA	2.206 to 27.56	0.0208
j (right)	Locomotion during stimulation	15 ct, 9 ea exp	one-way ANOVA, Dunnett's MC	F(2, 30) = 0.1622	0.0073	YFP vs. cCeA	-2.113 to 7.480	0.3450
						YFP vs. rCeA	2.209 to 11.80	0.0037
m	Area under curve CS responses	49 rostral, 22 caudal	2way RM, Sidak's MC	Interaction: F (1, 138) = 42.27	P<0.0001	CS 1: rCeA vs cCeA	-4.817 to 1.110	0.2937
				CS: F (1, 138) = 222.8	P<0.0001	CS 5: rCeA vs cCeA	-16.87 to -10.94	<0.0001
				Area: F (1, 138) = 72.27	P<0.0001			
o (top)	AFC TeTX rostral	8 ea.	2way RM ANOVA, Sidak's MC	Interaction: F (7, 98) = 25.33	P<0.0001	CS 1	-14.23 to 9.382	0.9847
				Time: F (7, 98) = 49.88	P<0.0001	CS 2	-24.83 to -1.218	0.0209
				Group: F (1, 14) = 262.1	P<0.0001	CS 3	-40.42 to -16.81	<0.0001
						CS 4	-43.03 to -19.42	<0.0001
						CS 5	-52.12 to -28.51	<0.0001
						CS 6	-62.01 to -38.39	<0.0001
						CS 7	-65.37 to -41.76	<0.0001
						CS 8	-69.74 to -46.13	<0.0001
o (right)	AFC TeTx caudal	8 ea.	2way RM ANOVA, Sidak's MC	Interaction: F (7, 98) = 27.50	P<0.0001	CS 1	-11.34 to 14.54	0.8542
				Time: F (7, 98) = 49.57	P<0.0001	CS 2	-23.14 to 2.739	0.3157
				Group: F (1, 14) = 121.3	P<0.0001	CS 3	-41.29 to -15.41	<0.0001
						CS 4	-39.50 to -13.62	<0.0001
						CS 5	-54.76 to -28.89	<0.0001
						CS 6	-56.56 to -30.69	<0.0001
						CS 7	-67.21 to -41.34	<0.0001
						CS 8	-67.53 to -41.65	<0.0001
p	TeTx cued fear recall	8 ea	one-way ANOVA, Dunnett's MC	F (2, 21) = 39.21	P<0.0001	YFP vs rCeA	31.42 to 59.95	<0.0001
						YFP vs cCeA	32.34 to 60.86	<0.0001

Figure 6								
Panel	Experiment	N (ct/exp)	Test	t/F (DFn, DFd)	P-values	Post-test	95% CIs	P-values
b	Proportion CS responsive		Bootstrapped CIs, 2way ANOVA, Sidak's MC	Interaction: F (2, 969) = 70.02	P<0.0001	Proportion excited: rCeA vs cCeA	-0.1127 to -0.06581	<0.0001

				Area: F (1, 969) = 3.472e-009	P>0.9999	Proportion no response: rCeA vs cCeA	0.04855 to 0.09543	<0.0001
				Response: F (2, 969) = 2917	P<0.0001			
c (right)	CS excited	37 caudal, 25 rostral	2way RM ANOVA, Tukey's MC	Interaction: F (2, 180) = 5.548	P=0.0046	Caudal Pre vs. CS	-5.386 to -2.543	<0.0001
				Time: F (2, 180) = 34.10	P<0.0001	Caudal Pre vs. Post	-4.519 to -1.676	<0.0001
				Group: F (1, 180) = 5.624	P=0.0188	Rostral Pre vs. CS	-4.005 to -0.5455	0.0073
						Rostral Pre vs. Post	-6.482 to -3.023	<0.0001
						Post: Caudal vs. Rostral	-4.350 to -0.9275	0.0008
d	Proportion freezing responsive	72 caudal, 59 rostral	Bootstrapped CIs, 2way ANOVA, Sidak's MC	Interaction: F (2, 969) = 455.8	P<0.0001	Proportion excited: rCeA vs cCeA	-0.2900 to -0.2500	<0.0001
				Area: F (1, 969) = 0.000	P>0.9999	Proportion inhibited: rCeA vs cCeA	0.3000 to 0.3400	<0.0001
				Response: F (2, 969) = 3713	P<0.0001	Proportion no response: rCeA vs cCeA	0.5700 to 0.6100	<0.0001
e	Cued freezing activated	72 caudal, 59 rostral	2way RM ANOVA, Sidak's MC	Interaction: F (1, 258) = 1.209	P=0.2726	Caudal: Pre vs. Post	-5.463 to -4.614	<0.0001
				Time: F (1, 258) = 1206	P<0.0001	Rostral: Pre vs. Post	-5.198 to -4.260	<0.0001
				Group: F (1, 258) = 0.01661	P=0.8976			
f	Cued freezing exc, CS response	72 caudal, 59 rostral	2way RM ANOVA, Sidak's MC	Interaction: F (2, 258) = 8.358	P=0.0003	Caudal: BL vs. CS	-2.982 to -0.6879	0.0008
				Time: F (1.803, 232.6) = 11.21	P<0.0001	Caudal: BL vs. Post	-2.440 to 0.3210	0.1650
				Group: F (1, 129) = 15.04	P=0.0002	Caudal: BL vs. Post	-0.2459 to 1.797	0.1712
						Rostral: BL vs. CS	-1.546 to 1.113	0.9192
						Rostral: BL vs. Post	-4.346 to -0.9420	0.0012
						Rostral: BL vs. Post	-3.817 to -1.039	0.0003
						BL: Caudal vs. Rostral	-2.027 to 0.8534	0.6936
						CS: Caudal vs. Rostral	0.4733 to 1.590	<0.0001
						Post: Caudal vs. Rostral	-3.473 to -0.8701	0.0003
h	Cued vs. Uncued auc	72 caudal, 59 rostral	2way RM ANOVA, Sidak's MC	Interaction :F (1, 129) = 1.094	P=0.2975	cCeA: cued uncued	1.169 to 2.647	<0.0001
				Freezing: F (1, 129) = 79.04	P<0.0001	rCeA: cued uncued	1.601 to 3.233	<0.0001
				Region: F (1, 129) = 2.721	P=0.1015			
i	CS activity no freezing	59 rostral	One sample Wilcoxan rCeA					
		72 caudal	One sample Wilcoxan cCeA	W: 1008	p=0.0043			
				W: 498.0	p=0.0671			
g	Freezing during stim	15 ct, 9 ea exp	2way RM ANOVA, Tukey's MC	Interaction: F (28, 420) = 23.05	P<0.0001	Stim 1: YFP vs cCeA	-32.38 to -20.19	<0.0001
				Time: F (6.195, 185.8) = 27.99	P<0.0001	Stim 1: YFP vs rCeA	-4.194 to 3.484	0.9680
				Group: F (2, 30) = 64.76	P<0.0001	Stim 1: cCeA vs rCeA	19.47 to 32.39	<0.0001
						Poststim 1: YFP vs cCeA	-21.98 to -0.8082	0.0361
						Poststim 1: YFP vs rCeA	-54.50 to -24.14	0.0002
						Poststim 1: cCeA vs rCeA	-44.81 to -11.05	0.0018
						Stim 2: YFP vs cCeA	-38.21 to -20.90	<0.0001
						Stim 2: YFP vs rCeA	-12.91 to 4.019	0.3448
						Stim 2: cCeA vs rCeA	14.20 to 36.02	<0.0001
						Poststim 2: YFP vs cCeA	-19.72 to 0.7905	0.0696
						Poststim 2: YFP vs rCeA	-61.46 to -21.33	0.0009
						Poststim 2: cCeA vs rCeA	-52.97 to -10.89	0.0043
						Stim 3: YFP vs cCeA	-38.25 to -21.58	<0.0001
						Stim 3: YFP vs rCeA	-6.046 to -0.2927	0.0292
						Stim 3: cCeA vs rCeA	18.42 to 35.07	<0.0001
						Poststim 3: YFP vs cCeA	-33.41 to -2.988	0.0219
						Poststim 3: YFP vs rCeA	-45.33 to -23.24	<0.0001
						Poststim 3: cCeA vs rCeA	-33.19 to 1.016	0.0666
m	Latency run or freeze stimulation	9 ea	2way RM ANOVA, Sidak's MC	Interaction: F (1, 36) = 77.37	<0.0001	Locomote: cCeA vs rCeA	2.439 to 12.49	0.0012
				Behavior: F (1, 36) = 39.99	<0.0001	Freeze: cCeA vs rCeA	-20.02 to -9.967	<0.0001
				Area: F (1, 36) = 8.695	0.0056	rCeA: Locomote vs Freeze	-24.07 to -14.53	<0.0001
p (right)	Conditioned heart rate GtACR2	3, 5 caudal, 6 rostral	Kruskal-Wallis, Dunn's MC	Kruskal-Wallis statistic: 8.517	0.0141	YFP vs. cCeA	NA	0.0076
						YFP vs. rCeA	NA	0.2651
q	Conditioned respiration GtACR2	3, 5 caudal, 6 rostral	one-way ANOVA, Dunnett's MC	F (2, 23) = 3.675	P=0.0412	YFP vs. cCeA	4.236 to 76.21	0.0278
						YFP vs. rCeA	-18.52 to 48.11	0.4708

Extended data Fig. 1							
Panel	Experiment	N (ct/exp)	Test	Comparison	R-square	95% CIs	P-values
e	PBN to CeA connectivity by number	12	Linear regression		0.05	-0.01487 to 0.1478	0.508
g	PBN to CeA connectivity AP -1.6	12	Linear regression		0.56	0.1047 to 0.8416	0.006

Extended data Fig. 2								
Panel	Experiment	N (ct/exp)	Test	t/F (DFn, DFd)	P-values	Post-test	95% CIs	P-values
b	rostral vs. caudal RNA *Slc32a1* +	6, 6	2way ANOVA, Sidak's MC	Interaction: F (7, 80) = 36.26	<0.0001	rostral-caudal: Calcrl	-13.67 to -0.3232	0.0342
				Gene: F (7, 80) = 61.32	<0.0001	rostra-caudal: Drd2	12.16 to 25.51	<0.0001
				Area: F (1, 80) = 18.46	<0.0001	rostral-caudal: Prkcd	-35.87 to -22.53	<0.0001
						rostral-caudal: Tacr1	-23.28 to -9.931	<0.0001
c	rostral vs. caudal RNA *Calcrl* +	6, 6	2way ANOVA, Sidak's MC	Interaction: F (6, 70) = 36.22	<0.0001	rostra-caudal: Drd2	16.47 to 36.16	<0.0001
				Gene: F (6, 70) = 72.84	<0.0001	rostral-caudal: Prkcd	-51.98 to -32.30	<0.0001
				Area: F (1, 70) = 30.09	<0.0001	rostral-caudal: Tacr1	-34.46 to -14.77	<0.0001

Extended data Fig. 4							
Panel	Experiment	N (ct/exp)	Test	Comparison	R-square	95% CIs	P-values
c	Expression/tracing correlations	10	Linear regression	CeL vs. SI	0.411	-0.01487 to -0.0001810	0.0458
				CeC vs. SI	0.5466	0.0006891 to 0.004665	0.0145
				CeA vs. SI	0.2894	-0.0005373 to 0.004407	0.1087
				Bias vs. SI	0.6721	-0.5022 to -0.1378	0.0037
d	Expression/tracing correlations	10	Linear regression	CeL vs. vBST	0.7639	0.002439 to 0.006485	0.0009
				CeC vs. vBST	0.01717	-0.001067 to 0.001479	0.7182
				CeA vs. vBST	0.1584	-0.0005475 to 0.001793	0.2547
				Bias vs. vBST	0.3829	-0.003682 to 0.2138	0.0565

Extended data Fig. 5								
Panel	Experiment	N (ct/exp)	Test	Comparison	R-square	95% CIs	P-values	
f	Excited rate of change	178 rostral, 166 caudal	Bootstrapped CIs; 2-way ANOVA, Sidak's MC	Interaction: F (2, 21) = 38.13	P<0.0001	Excited pos slope	-0.3251 to -0.1492	
				Area: F (1, 21) = 1.420e-008	P>0.9999	Excited neg slope	-0.01040 to 0.1655	
				Slope: F (2, 21) = 50.99	P<0.0001	Excited no slope	0.07161 to 0.2475	
g	slope	10000 bootstrap dist	t-test			0.00314 to -0.002145	0.2195	
h	improvement	10000 bootstrap dist	t-test			0.0318 to 0.141	0.0145	
i	sigmoid k	10000 bootstrap dist	t-test			20.12 to 129.76	0.007	

Extended data Fig. 6								
Panel	Experiment	N (ct/exp)	Test	Comparison	R-square	95% CIs	P-values	
b	Cell expression across CeA	8 ea.	2way ANOVA, Sidak's MC	Interaction: F (6, 98) = 40.53	P<0.0001	AP -2: Rostral vs. Caudal	15.94 to 83.81	0.0008
				AP level: F (6, 98) = 23.26	P<0.0001	AP -1.82: Rostral vs. Caudal	45.82 to 113.7	<0.0001
				Group: F (1, 98) = 5.258	P=0.0240	AP -1.64: Rostral vs. Caudal	42.94 to 110.8	<0.0001
						AP -1.46: Rostral vs. Caudal	-64.18 to 3.681	0.1091
						AP -1.28: Rostral vs. Caudal	-129.3 to -61.44	<0.0001
						AP -1.1: Rostral vs. Caudal	-136.1 to -68.19	<0.0001
						AP -0.92: Rostral vs. Caudal	-87.81 to -19.94	0.0002
c	Cell counts area comparison	8 ea.	2way ANOVA, Sidak's MC	Interaction: F (2, 42) = 9.991	P=0.0003	CeAl: Caudal vs. Rostral	-10.98 to 198.2	0.0915
				Group: F (1, 42) = 4.252	P=0.0454	CeAc: Caudal vs. Rostral	-273.4 to -64.14	0.0007
				CeA area: F (2, 42) = 86.19	P<0.0001	CeA: Caudal vs. Rostral	-179.7 to 29.48	0.2247
d	Expression/jumps correlations TeTx	16	Linear regression	CeL vs. Jumps	R2=0.1301		-0.004123 to 0.02122	0.1699
				CeC vs. Jumps	R2=0.5497		-0.01374 to -0.004353	0.0010
				CeA vs. Jumps	R2=0.3307		-0.01318 to -0.001339	0.0198
				Bias vs. Jumps	R2=0.4787		0.6045 to 2.404	0.0030
e	Paw-lick latency TeTx	8 ea.	one-way ANOVA, Tukey's MC	F (2, 21) = 1.500	P=0.2462			
f	Cumulative wall rearing TeTx	8 ea.	2way RM ANOVA, Tukey's MC	Interaction: F (118, 1180) = 4.054	P<0.0001			
				Time: F (1.690, 33.80) = 50.93	P<0.0001			
				Group: F (2, 20) = 3.546	P=0.0481			

Extended data Fig. 7									
Panel	Experiment	N (ct/exp)	Test	Comparison	R-square	95% CIs	P-values		
c	Paw-lick latency stim	5 ct, 5 caudal 4 rostral	one-way ANOVA, Tukey's MC	F (2, 11) = 17.08	P=0.0004	YFP vs. cCeA	-14.73 to 0.2547		0.0584
						YFP vs. rCeA	-25.13 to -9.243		0.0003
						cCeA vs. rCeA	-17.90 to -2.007		0.0155
d	Cumulative paw licking, stim	5 ct, 5 caudal 4 rostral	2way RM ANOVA, Tukey's MC	Interaction: F (226, 1243) = 8.283	P<0.0001				
				Time: F (1.439, 15.83) = 65.78	P<0.0001				
				Group: F (2, 11) = 11.36	P=0.0021				
f	Tail flick latency, stim	10 ct, 4 caudal, 5 rostral.	one-way ANOVA, Tukey's MC	F (2, 16) = 7.482	P=0.0051	YFP vs. cCeA	0.1334 to 1.080		0.0117
						YFP vs. rCeA	0.05904 to 0.9350		0.0252
						cCeA vs. rCeA	-0.6459 to 0.4269		0.8595
g (left)	Locomotion		2way ANOVA, Sidak's MC	Interaction: F (2, 32) = 15.33	P<0.0001	YFP: 52 vs 57	-40.92 to -14.42	<0.0001	
				Temperature: F (1, 32) = 7.924	P=0.0083	cCeA: 52 vs 57	-26.00 to -0.1374		0.0470
				Group: F (2, 32) = 0.7193	P=0.4948	rCeA: 52 vs 57	0.4104 to 28.88		0.0422
g (sec left)	Jumping		2way ANOVA, Sidak's MC	Interaction: F (2, 32) = 17.50	P<0.0001	YFP: 52 vs 57	-0.1323 to -0.01106		0.0163
				Temperature: F (1, 32) = 0.1161	P=0.7356	cCeA: 52 vs 57	-0.09803 to 0.02025		0.2888
				Group: F (2, 32) = 3.776	P=0.0337	rCeA: 52 vs 57	0.05990 to 0.1901	<0.0001	
g (sec right)	Paw licking		2way ANOVA, Sidak's MC	Interaction: F (2, 32) = 4.436	P=0.0199	YFP: 52 vs 57	-12.61 to 3.690		0.4438
				Temperature: F (1, 32) = 40.78	P<0.0001	cCeA: 52 vs 57	-21.78 to -5.869		0.0004
				Group: F (2, 32) = 5.162	P=0.0114	rCeA: 52 vs 57	-26.88 to -9.374	<0.0001	
g (right)	Wall rearing		2way ANOVA, Sidak's MC	Interaction: F (2, 32) = 6.488	P=0.0043	YFP: 52 vs 57	1.398 to 10.59		0.0074
				Temperature: F (1, 32) = 4.521	P=0.0413	cCeA: 52 vs 57	-0.4180 to 8.553		0.0848
				Group: F (2, 32) = 0.2290	P=0.7966	rCeA: 52 vs 57	-8.163 to 1.712		0.2941

Extended data Fig. 8									
Panel	Experiment	N (ct/exp)	Test	Comparison	R-square	95% CIs	P-values		
a	Stim induced anorexia	15, 8 caudal, 9 rostral	one-way ANOVA, Tukey's MC	F (2, 29) = 25.97	P<0.0001	YFP vs. cCeA	0.8327 to 1.961	<0.0001	
						YFP vs. rCeA	0.7254 to 1.812	<0.0001	
						cCeA vs. rCeA	-0.7546 to 0.4982		0.8693
b	Stim induced adipsia	15, 8 caudal, 9 rostral	Kruskal-Wallis test, Dunn's MC	Kruskal-Wallis statistic: 23.23	<0.0001	YFP vs. cCeA	15.69		0.0004
						YFP vs. rCeA	16.28		0.0001
						cCeA vs. rCeA	0.5903	>0.9999	
c	TeTx O/N food intake	8 ea.	One-way ANOVA	F (2, 21) = 0.5279	P=0.5975				
d	TeTx rehydration	8 ea.	One-way ANOVA	F (2, 21) = 1.892	P=0.1755				
e	TeTx meal size	8 ea.	Kruskal-Wallis test	Kruskal-Wallis statistic: 2.279	0.32				
f	TeTx meal number	8 ea.	One-way ANOVA	F (2, 21) = 1.700	P=0.2068				
g	TeTx meal duration	8 ea.	One-way ANOVA	F (2, 21) = 0.4492	P=0.6441				

Extended data Fig. 9									
Panel	Experiment	N (ct/exp)	Test	Comparison	R-square	95% CIs	P-values		
a	Distance moved	15, 8 caudal, 9 rostral	one-way ANOVA, Tukey's MC	F (2, 29) = 25.97	P<0.0001	YFP vs. cCeA	0.8327 to 1.961	<0.0001	
						YFP vs. rCeA	0.7254 to 1.812	<0.0001	
						cCeA vs. rCeA	-0.7546 to 0.4982		0.8693
c	Smoothed crossing latency rCeA	4	linear regression last 80 trials	Slope: -0.0174; Int: 7.37; R-sq: 0.379	<0.0001		-0.02240 to -0.01243		
	Smoothed crossing latency cCeA	5	linear regression last 80 trials	Slope: -0.0243; Int: 7.49; R-sq: 0.644	<0.0001	Slopes different? F(2, 158)=4.509, P=0.0353	-0.02830 to -0.02022		

Extended data Fig. 10									
Panel	Experiment	N (ct/exp)	Test	Comparison	R-square	95% CIs	P-values		
e	TeTx contextual fear	8 ea.	one-way ANOVA, Sidak's MC	F (2, 21) = 107.2	P<0.0001	YFP vs. cCeA	21.01 to 30.71	<0.0001	
						YFP vs. rCeA	15.87 to 25.57	<0.0001	
						cCeA vs. rCeA	-9.998 to -0.2965		0.0354
f	TeTx expression/conditioned fear	16	Linear regression	CeL vs. Freezing AUC	0.03487		-0.5159 to 0.2589		0.4886
				CeC vs. Freezing AUC	0.1601		-0.3278 to 0.04432		0.1246
				CeA vs. Freezing AUC	0.2637		-0.3684 to -0.007956		0.0419
				Bias vs. Freezing AUC	0.0239		-25.98 to 45.49		0.5676
j	GtACR2 inhibition	10, 6 rostral 5 caudal	two-way RM ANOVA	Interaction: F(14, 126) = 1161 1.193			p = 0.2888		

Extended data Fig. 11									
Panel	Experiment	N (ct/exp)	Test	Comparison	R-square	95% CIs	P-values		
a	Lens DV vs. conditioned freezing	9	Linear regression		0.01	-122.6 to 98.87	0.802		
c	Uncued freezing response	60 rCeA, 72 cCeA	2way RM ANOVA, Tukey's MC	Interaction: F (1, 130) = 5.326	P=0.0226	pre:post rCeA: -0.5330 to 1.247	0.5979		
				Time: F (1, 130) = 0.9361	P=0.3351	pre:post cCeA: -1.684 to -0.05975	0.0327		
				Region: F (1, 130) = 0.5297	P=0.4680				
d	Cued freezing response	92 rCeA, 61 cCeA	2way RM ANOVA, Tukey's MC	Interaction: F (1, 151) = 24.65	P<0.0001	pre:post rCeA: 0.7263 to 1.884	<0.0001		
				Time: F (1, 151) = 2.149	P=0.1447	pre:post cCeA: -1.421 to 0.0008639	0.0503		
				Region: F (1, 151) = 0.1135	P=0.7367				
e	Bootstrap CIs		2way ANOVA	Interaction: F (1, 14) = 53.22	P<0.0001				
				Region: F (1, 14) = 0.000	P>0.999				
				Response: F (1, 14) = 80.07	P<0.0001				

Supplementary Table 2

		Acronym	
CTXsp		PA	Posterior amygdalar nucleus
		LA	Lateral amygdalar nucleus
		EPv	Endopiriform nucleus, ventral part
		EPd	Endopiriform nucleus, dorsal part
		BLAa	Basolateral amygdalar nucleus, anterior part
		BLAp	Basolateral amygdalar nucleus, posterior part
		BLAv	Basolateral amygdalar nucleus, ventral part
		BMAa	Basomedial amygdalar nucleus, anterior part
		BMAp	Basomedial amygdalar nucleus, posterior part
CTXpl	HPC	DG	Dentate Gyrus
		SUB	Subiculum
		ENT	Entorhinal area
	OLF	PIR	Piriform area
		TR	Postpiriform transition area
		PAA	Piriform-amygdalar area
		NLOT	Nucleus of the lateral olfactory tract
		COAa	Cortical amygdalar area, anterior part
		COAp	Cortical amygdalar area, posterior part
		VISC	Visceral area
		GU	Gustatory area
		PERI	Perirhinal area
		TEa	Temporal association area
		ECT	Ectorhinal area
		AUDd	Dorsal auditory area
		AIp	Agranular insular area, posterior part
		SSs	Supplemental somatosensory area
PAL		SI	Substantia innominata
STR		BSTa	Bed nucleus of the stria terminalis, anterior division
		BSTal	Bed nucleus of the stria terminalis, anterolateral division
		CP	Caudoputamen
		AST	Amydala-striatal transition zone
		IA	Intercalated amygdalar nucleus
		AAA	Anterior amygdalar area
		MEAad	Medial amygdlara nucleus, anterodorsal part
		CeM	Central amygdalar nucleus, medial part
		CeC	Central amygdalar nucleus, capsular part
		CeL	Central amygdalar nucleus, lateral part
P		PBl	Parabrachial nucleus, lateral division
TH		PVT	Paraventricular nucleus of the thalamus
		SPFp	Subparafascicular nucleus, parvicellular part
		MG	Medial geniculate complex
		PP	Peripeduncular thalamic nucleus
		PIN	Posterior intralaminar thalamic nucleus
HY		LHA	Lateral hypothalamic area
		PSTN	Parasubthalamic nucleus

Macrostructure	
CTXsp	Cortical subplate
CTXpl	Cortical plate
PAL	Pallidum
STR	Striatum
P	Pons
TH	Thalamus
HY	Hypothalamus
MB	Midbrain
HPC	Hippocampal formation
OLF	Olfactory areas
