# Supplementary material for: Topographic representation of current and future threats in the mouse nociceptive amygdala
Source: Nat Commun. 2023 Jan 13;14:196. doi: 10.1038/s41467-023-35826-4 (PMC9839702; doi:10.1038/s41467-023-35826-4)
Supplement: Supplementary file 3 — Reporting Summary [file 41467_2023_35826_MOESM3_ESM.pdf]

nature portfolio

Corresponding author(s): Anna J Bowen
Richard D Palmiter

Last updated by author(s): Dec 12, 2022

Reporting Summary

Nature Portfolio wishes to improve the reproducibility of the work that we publish. This form provides structure for consistency and transparency in reporting. For further information on Nature Portfolio policies, see our Editorial Policies and the Editorial Policy Checklist.

Statistics

For all statistical analyses, confirm that the following items are present in the figure legend, table legend, main text, or Methods section.

n/a	Confirmed	
☐	☒	The exact sample size (*n*) for each experimental group/condition, given as a discrete number and unit of measurement
☐	☒	A statement on whether measurements were taken from distinct samples or whether the same sample was measured repeatedly
☐	☒	The statistical test(s) used AND whether they are one- or two-sided<br>*Only common tests should be described solely by name; describe more complex techniques in the Methods section.*
☐	☒	A description of all covariates tested
☐	☒	A description of any assumptions or corrections, such as tests of normality and adjustment for multiple comparisons
☐	☒	A full description of the statistical parameters including central tendency (e.g. means) or other basic estimates (e.g. regression coefficient) AND variation (e.g. standard deviation) or associated estimates of uncertainty (e.g. confidence intervals)
☐	☒	For null hypothesis testing, the test statistic (e.g. *F*, *t*, *r*) with confidence intervals, effect sizes, degrees of freedom and *P* value noted<br>*Give P values as exact values whenever suitable.*
☒	☐	For Bayesian analysis, information on the choice of priors and Markov chain Monte Carlo settings
☐	☒	For hierarchical and complex designs, identification of the appropriate level for tests and full reporting of outcomes
☒	☐	Estimates of effect sizes (e.g. Cohen's *d*, Pearson's *r*), indicating how they were calculated

Our web collection on statistics for biologists contains articles on many of the points above.

Software and code

Policy information about availability of computer code

Data collection	MouseOx Plus Conscious Software (1.5.31) was used for collection of autonomic data. Ethovision XT10 was used for video tracking of mouse behavior. Electrophysiology measurements were collected using pCLAMP 11.0.3 software (Molecular Devices). 1-photon calcium imaging was conducted using Inscopix3.0 software.
Data analysis	Image registration to atlas and cell segmentation were done in R 3.5.2 using publicly available Wholebrain algorithms. 1-photon cell segmentation and signal extraction was accomplished with publicly available MIN1PIPE software package run on MATLAB R2019a. GraphPad Prism 8.0 was used for most graphing and statistical testing. We used Python 3.8.0 and available libraries to write custom scripts for categorizing, analyzing, and plotting individual cell responses for calcium imaging data.

For manuscripts utilizing custom algorithms or software that are central to the research but not yet described in published literature, software must be made available to editors and reviewers. We strongly encourage code deposition in a community repository (e.g. GitHub). See the Nature Portfolio guidelines for submitting code & software for further information.

Data

Policy information about availability of data

All manuscripts must include a data availability statement. This statement should provide the following information, where applicable:

- Accession codes, unique identifiers, or web links for publicly available datasets
- A description of any restrictions on data availability
- For clinical datasets or third party data, please ensure that the statement adheres to our policy

Data generated in this study are provided in the Source Data file.

Field-specific reporting

Please select the one below that is the best fit for your research. If you are not sure, read the appropriate sections before making your selection.

☒ Life sciences ☐ Behavioural & social sciences ☐ Ecological, evolutionary & environmental sciences

For a reference copy of the document with all sections, see nature.com/documents/nr-reporting-summary-flat.pdf

Life sciences study design

All studies must disclose on these points even when the disclosure is negative.

Sample size	No sample-size calculations were performed. The sample size was based on prior publications using similar behavioral paradigms (Han et al. 2016, Campos et al. 2018, Bowen et al. 2020).
Data exclusions	Individual subjects were excluded from analysis when post-hoc analysis of viral expression and/or fiber placement revealed low expression or off-target fiber placement.
Replication	Calcium imaging, anterograde and retrograde tracing, and behavioral experiments involving excitation were successfully repeated in two groups. Permanent inhibition, slice electrophysiology and RNAscope experiments include data from single groups of mice.
Randomization	Mice from each litter were randomly assigned into treatment groups
Blinding	Investigators were not blinded to group allocation during data collection due to presence of subject identifiers on cage cards. Investigators were blinded to group allocation during data analysis.

Reporting for specific materials, systems and methods

We require information from authors about some types of materials, experimental systems and methods used in many studies. Here, indicate whether each material, system or method listed is relevant to your study. If you are not sure if a list item applies to your research, read the appropriate section before selecting a response.

Materials & experimental systems

n/a	Involved in the study	
☐	☒	Antibodies
☐	☒	Eukaryotic cell lines
☒	☐	Palaeontology and archaeology
☐	☒	Animals and other organisms
☒	☐	Human research participants
☒	☐	Clinical data
☒	☐	Dual use research of concern

Methods

n/a	Involved in the study	
☒	☐	ChIP-seq
☒	☐	Flow cytometry
☒	☐	MRI-based neuroimaging

Antibodies

Antibodies used	Chicken polyclonal anti-GFP, Abcam, Cat# ab13970, RRID: AB_300798; Rabbit monoclonal anti-dsRed, Takara Bio, Cat#632496, RRID: 10013483; Alexa Fluor 488 donkey anti-chicken, Jackson ImmunoResearch, Cat# 703-545-155, RRID: AB_2340375; Alexa Fluor 594 donkey anti-rabbit, Jackson ImmunoResearch ,Cat# 711-585-152, RRID: AB_2340621.
Validation	Chicken polyclonal anti-GFP validated by manufacturer for wholemount IHC of mouse tissue. Rabbit monoclonal anti-dsRed first validated in mice by Tseng et al. 2010 (PMID 20575070) and cited in over 225 peer reviewed articles since then.

Eukaryotic cell lines

Policy information about cell lines

Cell line source(s)	HEK 293 cells were sourced from a cell bank originally sourced from ATCC.
Authentication	HEK 293 cells were not authenticated.
Mycoplasma contamination	HEK 293 cells tested negative for mycoplasma contamination.
Commonly misidentified lines (See ICLAC register)	*Name any commonly misidentified cell lines used in the study and provide a rationale for their use.*

Animals and other organisms

Policy information about studies involving animals; ARRIVE guidelines recommended for reporting animal research

Laboratory animals	Mouse, C57BL6/J, both males and females aged 3-7 months. Calcrl-Cre heterozygous mice were generated and maintained as described (Han et al., 2015). Mice were housed with ad libitum access to standard chow diet (LabDiet 5053) in temperature- and humidity-controlled facilities (17-23°C and 40-50% humidity) with 12-h light/dark cycles.
Wild animals	The study did not involve wild animals.
Field-collected samples	The study did not involve collection of field samples.
Ethics oversight	Study protocol was approved by the IACUC at University of Washington.

Note that full information on the approval of the study protocol must also be provided in the manuscript.
